# Song in a Social and Sexual Context: Vocalizations Signal Identity and Rank in Both Sexes of a Cooperative Breeder

**DOI:** 10.3389/fevo.2016.00046

**Published:** 2016-05-03

**Authors:** Sara Keen, C. Daniel Meliza, July A. Pilowsky, Dustin R. Rubenstein

**Affiliations:** 1 Department of Neurobiology and Behavior, Cornell University, Ithaca, NY, USA; 2 Bioacoustics Research Program, Cornell Lab of Ornithology, Cornell University, Ithaca, NY, USA; 3 Department of Ecology, Evolution and Environmental Biology, Columbia University, New York, NY, USA; 4 Department of Psychology, University of Virginia, Charlottesville, VA, USA; 5 Department of Biology, Tufts University, Medford, MA, USA; 6 Center for Integrative Animal Behavior, Columbia University, New York, NY, USA

**Keywords:** female song, sexual selection, social selection, cooperative breeding, *Lamprotornis superbus*

## Abstract

In most songbirds, both sexes produce calls, or short vocalizations used to coordinate behaviors and maintain social cohesion. In contrast, songs are longer, more elaborate vocalizations typically only produced by males in behavioral contexts shaped by sexual selection operating through female choice. However, both males and females sing in many cooperatively breeding species, including the superb starling (*Lamprotornis superbus*). In this species, both sexes produce songs and calls composed of sequences of temporally discrete elements called motifs. Calls signal social group and individual identity, but the function of songs is currently unknown. Because superb starlings often sing in groups, song could be used not only in a sexual context, but also to signal identity and rank within the separate dominance hierarchies observed in males and females. To determine whether songs are used in mate attraction (sexually selected) and/or to influence social rank (socially selected), we compared song diversity with three potential indicators of fitness and dominance: social status, the number of seasons spent breeding, and age. We found that age is correlated with song diversity in both males and females, suggesting that (1) these signals serve similar purposes in both sexes, and (2) song diversity is likely the result of selection by both mutual mate choice and social competition. To test whether songs carry a signal of individuality, we applied spectrogram dynamic time warping to measure pairwise similarity among song motifs, and then calculated motif similarity within and between individuals. We found that motif similarity is higher within individuals than between individuals, suggesting that songs signal individual identity, which may help to establish social rank. These results are consistent with the hypothesis that superb starling vocal behavior in each sex is shaped by both social and sexual selection. Additionally, because call motifs are also used in songs, our data suggest that at least some vocal building blocks have evolved to convey multiple signaler traits and to facilitate complex social and sexual interactions in different contexts.

## INTRODUCTION

Bird song has traditionally been viewed either as a male ornament used to attract females or an armament used to defend territories against other males (Darwin, 1859). Under the traditional model of sexual selection (Darwin, 1871), song was thought to be an example of male trait elaboration resulting from differential selection pressures between the sexes due to female choice (Andersson, 1994). Female choice remains a common explanation for male song in temperate-dwelling species that have socially monogamous mating systems (Catchpole and Slater, 2008). However, recent studies have shown that a large proportion of avian species exhibit female song (Langmore, 1998; Garamszegi et al., 2007; Odom et al., 2014). For example, several studies have shown that female song frequently occurs in tropical species that occupy year-round territories (Morton, 1996; Langmore, 1998; Hall, 2004; Slater and Mann, 2004; Price, 2009; Price et al., 2009; Tobias et al., 2011), as well as in species where females may use song for territory defense (Cooney and Cockburn, 1995). In species where competition among females is high, it has recently been suggested that elaborate female traits are under sexual selection much in the same way as they are in males: acting through male mate choice and female-female competition (Clutton-Brock, 2007, 2009; Rubenstein and Lovette, 2009; Rubenstein, 2012b). There is also increasingly strong support for the idea that ornamentation in females can be influenced by social selection, or social competition for ecological resources that indirectly lead to an increased likelihood of reproducing (Crook, 1972; West-Eberhard, 1979, 1983; Lyon and Montgomerie, 2012; Tobias et al., 2012). Thus, there has been a renewed interest in female ornamentation and the recognition that we must develop models of selection that not only account for this mutual trait elaboration (Clutton-Brock, 2007; Tobias et al., 2011; Rubenstein, 2012a), but also reframe sexual selection theory to be more universally applicable to trait elaboration in both sexes (Jones and Ratterman, 2009; Price, 2015).

Among highly social birds, a suite of selective forces may drive patterns of trait elaboration in both sexes. In cooperatively breeding societies, for example, social selection for shared resources as well as sexual selection for mates may lead to ornamentation in both males and females because there is typically strong competition for limited breeding positions in each sex (Rubenstein and Lovette, 2009). Additionally, in societies where animals live in stable groups, dominance hierarchies often emerge (Clutton-Brock and Huchard, 2013), which could lead to selection for traits that aid in establishing social rank (reviewed in Tibbetts and Dale, 2007). In some cases, different features of a single trait may be shaped simultaneously by both sexual and social selection, such that the trait serves both to attract mates and to aid in competition for ecological resources and/or social rank. For example, in cooperatively breeding mockingbirds, song repertoire size appears to be driven by sexual selection operating though female choice (Howard, 1974), whereas song consistency has been shown to function in both a social and sexual context, signaling age, social dominance, and reproductive success in males (Botero et al., 2009). Similarly, in cooperatively breeding Malurids, the strength of sexual selection is correlated with singing rates in males of several species, but syllable diversity is determined by environmental factors correlated with latitude (Greig et al., 2013). Finally, simple vocalizations (e.g., calls) are under strong selection to signal individual, kin, or group identity in many cooperatively breeding birds (Payne et al., 1988; Price, 1998; McDonald and Wright, 2011), suggesting that vocal signals may also be shaped by the need to facilitate cooperation and competition in socially complex species. Signaling identity can help to establish rank or maintain dominance hierarchies (Barnard and Burk, 1979; Pagel and Dawkins, 1997), and the need to signal individual identity may therefore play a role in shaping songs in social species. Although it is widely accepted that song is constrained by multiple traits (Gil and Gahr, 2002), few studies have examined how sexual and social selection—including the need to signal identity—shape song evolution and the extent to which this occurs in both males and females. Furthermore, to our knowledge, no previous studies have examined calls and songs together to compare the relative strength of selection on these different types of vocal signals.

Here we investigate the calls and songs of male and female cooperatively breeding superb starlings (*Lamprotornis superbus*) to explore how sexual selection to attract or gain access to mates, as well as social selection to establish dominance rank, may influence trait evolution. Superb starlings are plural cooperative breeders that live in large social groups of up to 30 or more birds that can include as many as six breeding pairs (Rubenstein, 2016). Many non-breeders serve as helpers at the nest, and there is high intrasexual competition in both males and females for limited breeding positions (Rubenstein, 2007a). As in other African starlings that breed cooperatively, superb starlings show reduced sexual dimorphism in body size and plumage (Rubenstein and Lovette, 2009), and Bateman gradients are similar in males and females, further supporting the idea that both sexes are under strong sexual selection (Apakupakul and Rubenstein, 2015). This lack of sexual dimorphism extends to song as well, with both males and females producing similarly complex songs (Pilowsky and Rubenstein, 2013). Superb starling songs include a large number of unique motifs, or single notes that are arranged in various combinations (Pilowsky and Rubenstein, 2013). Starlings also produce short, relatively simple vocalizations (i.e., four or five motifs long) when flying over group territories. These flight calls have been shown to carry a strong signal of individual identity and social group membership, and are therefore thought to function in recognition (Keen et al., 2013). Interestingly, all of the motifs used in flight calls (hereafter, “calls”) also appear in songs, meaning that starlings take these basic vocal components and add many more song-specific motifs to create elaborate vocal displays when singing.

Superb starling breeders have been shown to produce more unique motifs in their songs than non-breeders (Pilowsky and Rubenstein, 2013), but exactly how song functions in a social and sexual context in this species remains unclear. We hypothesize that song elaboration is shaped by mutual mate choice (i.e., sexual selection in both sexes), and predict that song diversity is correlated with reproductive success in both males and females. Furthermore, because songs include many of the motifs used in flight calls, we hypothesize that song also functions in recognition, which may be important for establishing dominance rank. We predict that a signal of individual identity is embedded within a song, just as it is within a call. Consequently, we expect that song is a complex signal shaped by both sexual and social selection to signal fitness, social rank, and identity. To test these predictions, we analyzed song and call recordings collected from male and female superb starlings to determine (1) if song diversity is correlated with total lifetime breeding opportunities, age, or status, (2) if a signal of individual identity is present in songs and whether it is stronger than that in calls, and (3) if song diversity and individuality are equivalent in males and females.

## METHODS

### Study Population

All data were collected from a free-living population of superb starlings at the Mpala Research Centre, Laikipia, Kenya between May and July 2008–2011. This population has been studied continuously since 2001, and individuals have been marked with a unique combination of colored leg bands and a metal leg ring with an identification number (Rubenstein, 2007a). The study population includes nine social groups that maintain year-round territories. Male superb starlings are typically philopatric, whereas females immigrate after reaching maturity (Rubenstein, 2007a). However, within-group relatedness among males is lower than expected, as nearly half of all male breeders may be immigrants (Pollack and Rubenstein, 2015; Rubenstein, 2016). Additionally, relatedness among females is higher than expected, as immigrant females recruit sisters to their new groups (Pollack and Rubenstein, 2015). Thus, kin structure is present in both sexes within a group, though higher than expected in females and lower than expected in males (Rubenstein, 2016). All field work for this study was approved by Columbia University’s Institutional Animal Care and Use Committee (#ACAAAB1128).

### Song Data

Audio recordings of songs and calls were collected as part of previous studies (Keen et al., 2013; Meliza et al., 2013; Pilowsky and Rubenstein, 2013) using a Sennheiser ME66 directional microphone (Sennheiser Electronic, Old Lyme, CT) and a Marantz PMD661 digital recorder (Marantz, Mahwah, NJ). Audio files were saved as 16-bit, 44 kHz wav files and were automatically time-stamped upon recording. Focal birds were identified using a spotting scope, and age, breeding status, and the total number of seasons spent breeding were obtained from behavioral observations and long-term census records (Pollack and Rubenstein, 2015; Rubenstein, 2016).

**FIGURE 1 |:**
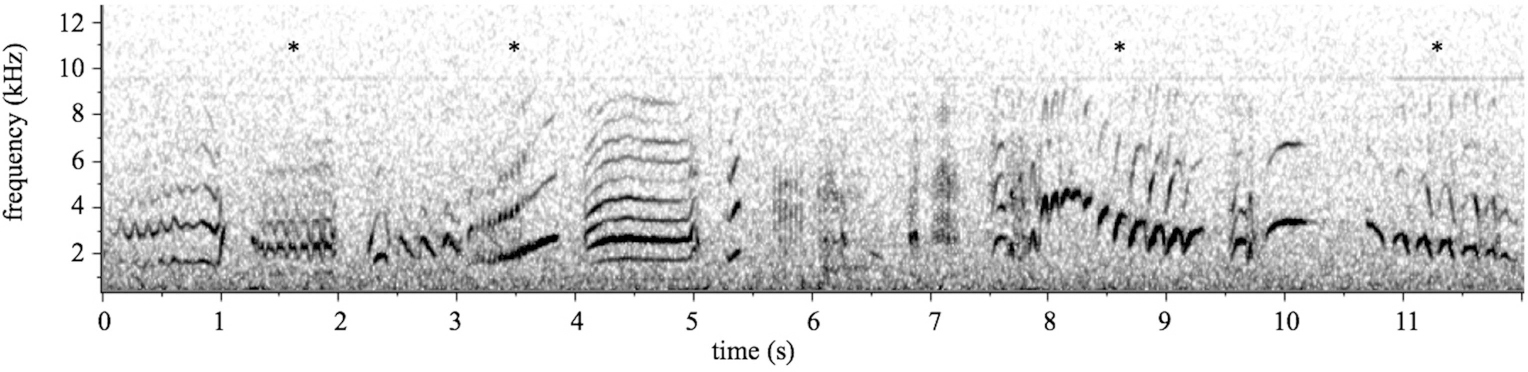
Spectrogram of superb starling song. Superb starling song is a complex signal comprising over 80 unique motifs used in various combinations. In contrast, superb starling calls are relatively simple combinations of up to 20 possible motifs, though a single call bout typically contains only 4–5 motifs. Notably, all of the motifs used in calls are also found in songs. This 11-s spectrogram of superb starling song includes several motifs that are also used in flight calls, indicated here by asterisks.

Songs, which we defined as vocalizations lasting more than 5 s, were recorded from 28 individuals (16 males and 12 females) in five social groups. In contrast, bouts of flight calls were much shorter and typically lasted between 0.5 and 2 s. Songs were collected from two individuals in 2008 and 26 individuals in 2011; songs were not collected from the same individual in multiple years. We showed previously that at least 8 min of song are necessary to assess a superb starling’s repertoire size (Pilowsky and Rubenstein, 2013). Therefore, we collected 8.8 ± 0.2 min (mean ± SD) of song from the 28 sampled individuals, and 26 of these individuals had over 8 min of song. Superb starlings sing only when perched, and during singing do not perform other behaviors except for occasional preening (Pilowsky and Rubenstein, 2013). All recordings were divided into individual wav files each containing a single song motif (*sensu* Pilowsky and Rubenstein, 2013), using Raven Pro 1.2 (Cornell Lab of Ornithology, Ithaca, NY, U.S.A.). Motifs were identified as single notes that are visible as continuous tonal elements in spectrograms and can be separated from adjacent motifs by the absence of any power between 1 and 12 kHz for at least 40 ms (see Figure 1). Motif categories were created during a previous study of song in this population (Pilowsky and Rubenstein, 2013; see data supplement for motif category key). This yielded a dataset of 20,423 song motifs (12,797 from males and 7626 from females), and 729 ± 249 motifs (mean ± SD) per individual. All song motifs were then manually labeled by J.A.P. as belonging to one of 87 distinct motif classes.

Flight call recordings were collected during 2008–2010 from 109 individuals (56 males and 53 females), including at least five birds in each of the nine social groups. Call recordings were divided into motifs in the same manner as songs, and were classified into 18 unique classes, all of which also appear in songs. After excluding recordings of poor quality or those in which the focal bird was unidentifiable, the final dataset contained 1936 call motifs (1095 from males and 841 from females), with 39 ± 19 motifs (mean ± SD) motifs per individual. Spectrograms depicting songs and calls are shown in Figure 1.

When recording both flight calls and songs, the microphone was always placed within 5–10 m of the focal bird, and the gain on the digital recorder was adjusted to ensure that the recording maximized the dynamic range of the digitizer but did not clip. As an additional means of accounting for differences in recording distance, the amplitude of all wav files of single motifs were normalized before any sound analysis was performed.

### Song Diversity Analyses

We calculated within-bird song motif diversity for the 28 individuals from which we had song recordings using the Shannon Diversity Index (H’) (Shannon and Weaver, 1949) of the 87 manually-assigned motif labels observed in the larger population, yielding a single measure of song diversity for each individual. The Shannon diversity index was selected because it accounts for both richness (i.e., the number of different types of motifs in an individual’s song) as well as evenness (i.e., their relative abundances), and has been shown to be the best metric for combining these components into a single estimate of diversity (Stirling and Wilsey, 2001). We used linear mixed-effects models to test whether age, status at time of recording, proportion of seasons spent breeding, sex, and social group were predictors of song diversity. Based on model comparisons using Akaike’s information criterion (AICc) (summarized in Table S1), we excluded interaction terms from the model. All of the predictors showed a low degree of multicollinearity (all VIF < 2.2). In our model, social group was used as a random effect; all other variables were included as fixed effects. Status at time of recording was recorded as “breeder” or “non-breeder,” which was determined through nest observations during the season in which songs were recorded. The proportion of seasons spent breeding was calculated as the total number of seasons during which an individual held “breeder” status, divided by the total number of seasons in which they were alive and capable of holding a breeding position (i.e., older than 1 year of age). We used this measurement rather than lifetime reproductive success (i.e., total number of offspring fledged) because high nest predation rates and unpredictable breeding conditions in this population make this an unreliable indicator of the number of breeding opportunities an individual obtained. Moreover, we know from previous work in this system that the number of seasons breeding is the strongest predictor of lifetime reproductive success (Apakupakul and Rubenstein, 2015). Thus, our selected predictor variables (i.e., age, current status, proportion of seasons breeding) are all believed to be correlated with fitness and dominance status in superb starlings.

### Song Similarity Analyses

To determine if songs carry a signal of individual or group identity, we used dynamic time warping (hereafter DTW) (Vintsyuk, 1971; Kogan and Margoliash, 1998) to compare spectrograms of individual song motifs to one another (See Table S2 for spectrograms of all motifs in our dataset). DTW quantifies the similarity of two spectrograms by compressing or expanding the reference spectrogram on the time axis in order to find the best fit (Vintsyuk, 1971). This method is less sensitive to background noise than spectrographic cross-correlation and produces similarity measures that more closely match human assessments of similarity between spectrograms of superb starling vocalizations (Meliza et al., 2013). The performance and repeatability of the DTW algorithm on call motifs in superb starlings was examined previously (Meliza et al., 2013); we also examined the algorithm’s concordance with song motif categories and found that average similarity scores within categories (mean ± SE = 2.57 ± 0.002) were significantly greater than scores between song motif categories (mean ± SE = 2.46 ± 0.0004; *t*-test: *t* = −60.5, *p* < 0.001).

We calculated similarity scores for all pairs of call and song motifs in the dataset using the pairwise distance metric output by the DTW analysis (*sensu* Meliza et al., 2013). We then identified the best match for each motif within the repertoire of every bird in the dataset, including the individual that sang the reference motif (Keen et al., 2013). This best match score is high if there is a close match with a motif in the target bird’s repertoire, but low if there is not. Best match scores were then grouped by whether the target bird was (1) the individual that sang the reference motif, (2) in the same social group, or (3) in a different social group. Call and song motifs were analyzed separately. We used a linear mixed-effects model (LMM) to test whether mean best match scores (log transformed for normality) depended upon social relationship (i.e., same bird, same group, or different group), with random effects included for the year the recording was collected, the sex, the social group of the birds whose motifs were being compared, and the reference motif used. The number of motifs tested for each comparison bird was also included as a covariate to account for differences in the number of samples per individual and the increased probability of finding a good match with larger numbers of comparisons.

To test whether within-bird similarity was higher within songs or calls, we used a Welch two sample *t*-test to compare mean within-bird song best match scores (*N* = 28) to mean within-bird call best match scores (*N* = 89). Similarly, we used a Welch two-sample *t*-test to compare whether mean within-bird call and song similarity was higher in males or females, using separate tests for calls and songs. In both cases Welch *t*-tests were used to account for unequal variances.

All statistical tests were conducted in R (R Development Core Team, 2015); mixed-effects models were fit using *lme4* (Bates et al., 2015), and the significance of fixed effects was evaluated using Satterthwaite approximations to estimate effective degrees of freedom. *Post-hoc* comparisons used Tukey’s tests to evaluate significance. Although this technique is nearly identical to that used in Keen et al. (2013) to analyze call motifs, song motifs were compared here using DTW of spectrograms rather than pitch traces. Pitch-based DTW is more sensitive than spectral DTW because it effectively eliminates background noise, though the results are qualitatively similar (Meliza et al., 2013).

## RESULTS

### Song Diversity Is Correlated with Age and Breeding Experience

**FIGURE 2 |:**
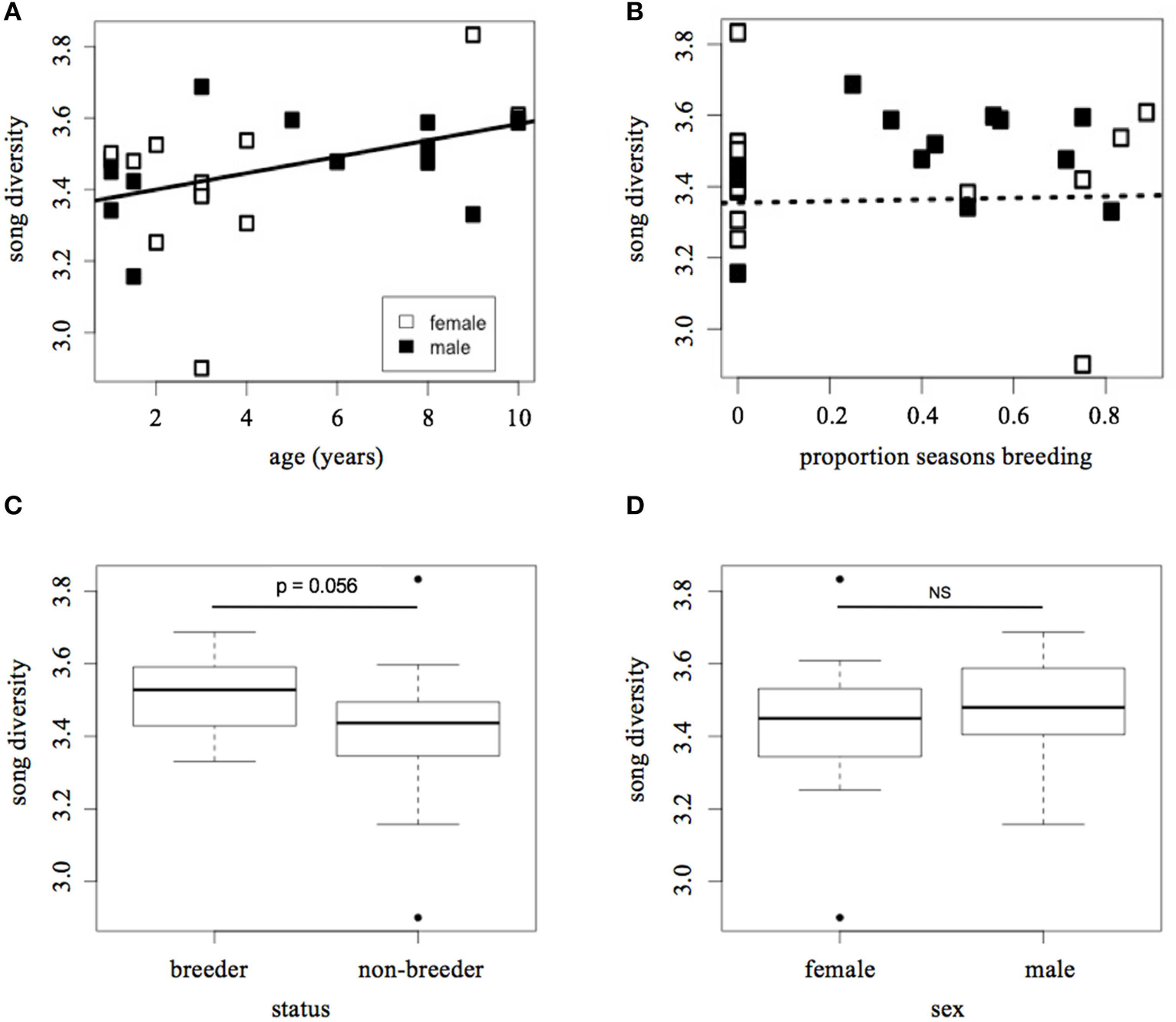
Song diversity vs. indicators of individual fitness and/or social dominance. **(A)** Song diversity vs. age, **(B)** song diversity vs. proportion of seasons spent breeding, **(C)** song diversity vs. social status, **(D)** song diversity vs. sex. Diversity is calculated using the Shannon Diversity Index (H′). Social status at time of recording (i.e., “breeder” or “non-breeder”) was determined from nest observations. Proportion of seasons breeding was measured as the total number of seasons during which an individual held “breeder” status, divided by the total number of seasons in which they were alive and capable of holding a breeding position (i.e., older than 1 year of age). Lines in **(A,B)** represent best fit; the dashed line indicates that the correlation in **(B)** is not statistically significant.

Song diversity increased with age (LMM: *t* = 2.37, *p* = 0.028; Figure 2A), but decreased with the proportion of seasons spent breeding (*t* = −2.49, *p* = 0.021). Although Figure 2B appears to show a positive relationship between song diversity and breeding experience, once the correlation with age is taken into account, the data indicate that more successful breeders tend to have less diverse song repertoires. However, there was a trend for breeders at the time of the recording to have more song diversity than helpers (LMM: *t* = 2.01, *p* = 0.056; Figure 2C). Males and females sang equally diverse songs (LMM: *t* = 0.40, *p* = 0.69; Figure 2D), and there was little variation among groups in song diversity relative to variation within groups (SD among groups = 0.057; residual SD = 0.15). These results are summarized in Table 1.

### Songs Carry a Signal of Individual Identity but Not Social Group

**FIGURE 3 |:**
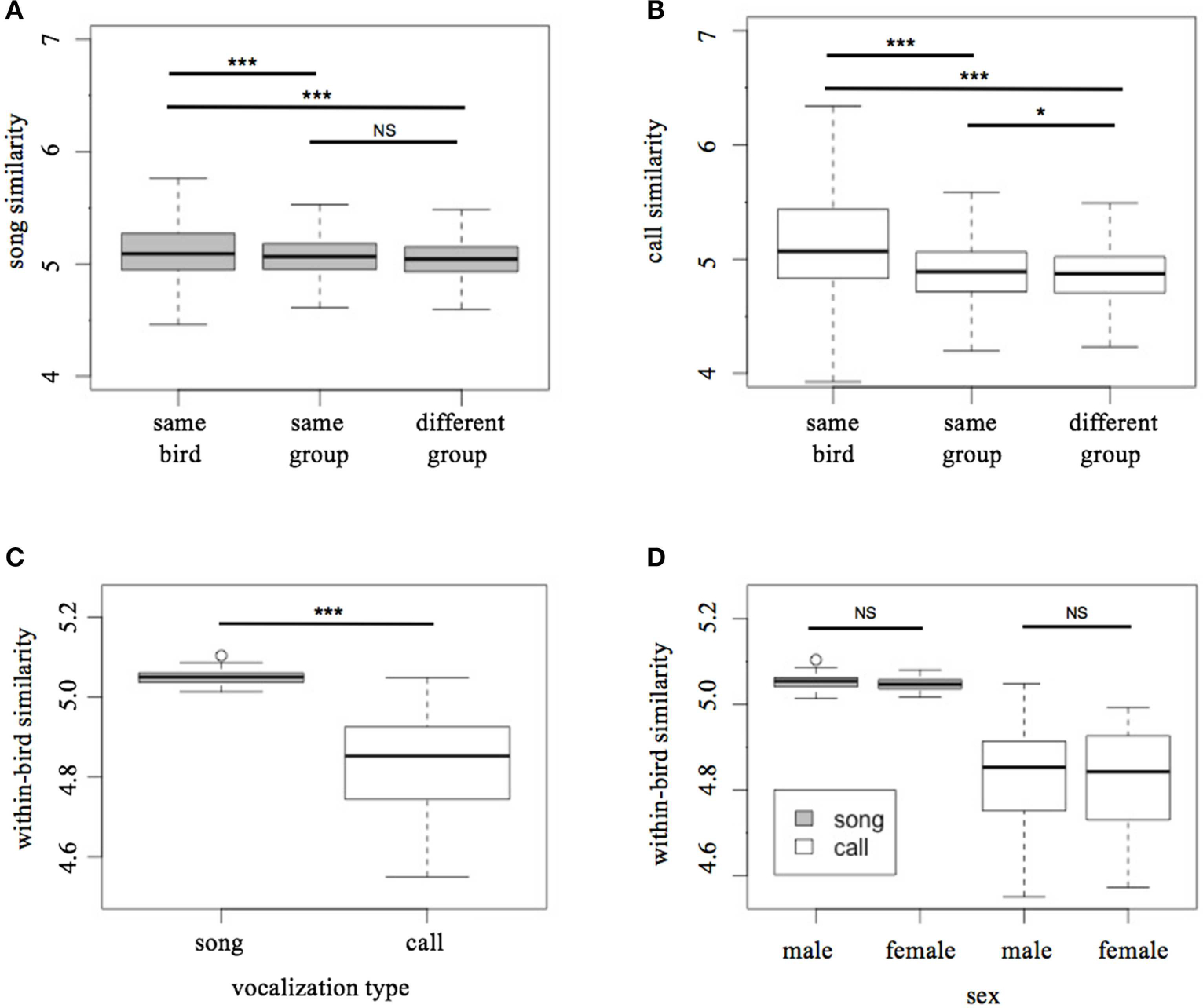
Patterns of similarity in songs and calls. **(A)** Song motif similarity vs. social relationship, **(B)** call motif similarity vs. social relationship, **(C)** within-bird motif similarity of songs and calls, **(D)** within-bird motif similarity of songs and calls with sexes included. Units for the y-axis are arbitrary. *p < 0.5, **p < 0.01, ***p < 0.001.

Song motifs from the same bird’s repertoire were significantly more similar to each other than to song motifs of birds in the same social group (LMM Tukey test: *t* = 47.4, *p* < 0.001; Figure 3A), as well as song motifs of birds in other social groups (*t* = 5.71, *p* < 0.001; Figure 3A). However, song motifs were not more likely to be similar to those of other birds in the same social group than to song motifs of those in other groups (*t* = 1.61, *p* = 0.21; Figure 3A).

As with song motifs, call motifs from the same bird’s song repertoire were significantly more similar to each other than to same-group birds (LMM Tukey test: *t* = 34.5, *p* < 0.001; Figure 3B) as well as to extra-group birds (*t* = 21.676, *p* < 0.001; Figure 3B). Unlike song, however, call motifs were more similar to calls from other individuals in the same social group than to those of birds in the larger population (*t* = 4.93, *p* < 0.05; Figure 3B).

### Differences Between Signal Types and Sexes

Within-bird similarity in songs was significantly higher than in calls (Welch two sample *t*-test: *t* = −13.96, *p* < 0.001; Figure 3C). In both songs and calls, males and females did not have significantly different levels of within-bird motif similarity (*t*-test: song, *t* = −0.75, *p* = 0.46; call, *t* = −0.57, *p* = 0.57; Figure 3D).

## DISCUSSION

Our results show that older birds sing more diverse songs, consistent with the hypothesis that song complexity is a signal of current status within superb starling social groups. Interestingly, individuals with more breeding experience tended to sing less diverse songs than individuals of the same age, suggesting that additional factors may influence the development of more complex songs. Moreover, song motif similarity was significantly higher when comparing motifs produced by the same individual than when comparing motifs produced by different birds. Together, these results suggest that superb starling song is likely to signals status and individual identity, and that song complexity may stabilize or decrease in older individuals once breeding positions are obtained. The observed equivalent degree of song diversity in males and females indicates that selection for elaborate songs may be equally strong in both sexes. This result is consistent with reproductive success data from this population showing that males and females are both likely to be under strong sexual selection for access to mates (Apakupakul and Rubenstein, 2015).

In studies of species with mating systems driven by female choice, older mates have often been shown to be preferred by females (Andersson, 1994; Kokko and Lindström, 1996; Kokko, 1997). The correlation between song elaboration and age in both male and female superb starlings could be similarly shaped by preference for older mates. Several other species have been shown to signal age with song complexity, particularly in other open-ended learners such as willow warblers (*Phylloscopus trochilus*; Gil et al., 2001), swamp sparrows (*Melospiza georgiana*; Ballentine, 2009), and European starlings (*Sturnus vulgaris*; Mountjoy and Lemon, 1995). Superb starlings can live for up to 12 years or more in the wild and breed twice annually (Rubenstein, 2006), meaning that individuals have numerous opportunities to compete for breeding positions during their lifetimes. Therefore, in obligate cooperative breeders like this one, older males could be preferred by immigrant females because they are more likely to have other offspring from previous breeding attempts to act as helpers (Rubenstein, 2006), which are important for increasing the likelihood of successfully fledging offspring (Rubenstein, 2007b). Rather than song complexity increasing with age, an alternative explanation could be that individuals with more complex songs have longer lifespans and are simply over-represented in our study. Additional work will be needed to determine if this is the case, as the data presented here are derived from a cross-sectional sample of the population and do not allow us to test this alternative hypothesis.

Although song diversity appears to increase as starlings become older, those individuals that obtain breeding positions less frequently have more diverse song, a result that contrasts with our initial prediction. This inconsistency may be due to breeders investing more energy in reproduction than in singing or developing more diverse songs. Alternately, breeders may not need to use song displays to compete for breeding positions, as helpers do, since after becoming a breeder, many starlings hold breeding positions for several subsequent seasons (Rubenstein, 2016). Although song diversity may not continue to increase as breeders age, the observed trend of older males and females having more diverse songs suggests that song elaboration is likely to be an honest indicator of social dominance.

Song diversity appears to be shaped by sexual selection in both males and females, but it is also likely to be under strong social selection. Social rank is a key determinant of whether superb starlings will obtain breeding opportunities, and older individuals typically outcompete others for limited breeding positions within social groups (Rubenstein, 2016). Thus, age may be a proxy for dominance status in this species, and a signal of age in song could facilitate social competition for shared resources while also helping to establish or maintain rank. The degree to which song diversity is driven by sexual vs. social selection is difficult to tease apart, as dominance status achieved through social competition leads to more breeding opportunities, thereby indirectly influencing fitness. The complexity of the relationship between social and sexual selection, particularly in the context of social rank or dominance status, has been highlighted in recent studies (Lyon and Montgomerie, 2012; Rubenstein, 2012b; Tobias et al., 2012; Clutton-Brock and Huchard, 2013). Although difficult to disentangle, our results are consistent with the hypothesis that song is used in both a sexual and social context in both sexes of superb starlings, as well as with previous work showing that mutual ornamentation can function in signaling dominance and in attracting mates in species with high social competition in both sexes (Kraaijeveld et al., 2004; Viera et al., 2008).

Interestingly, our findings differ from recent work showing that cooperatively breeding striped-headed sparrows (*Peaucaea ruficauda*) show role reversal in song, with females having larger repertoires than males (Illes, 2015), and with evidence of social selection acting more strongly on female song in cooperatively breeding superb fairy-wrens (*Malurus cyaneus*; Cain and Langmore, 2015). This difference may reflect the fact that females of these species experience stronger intrasexual competition for resources than males due to subtle differences in their social systems: striped-headed sparrows live in social groups composed mostly of males (Illes, 2015), and female fairy wrens typically disperse and join shared territories that they often help to defend during males’ frequent absences (Cooney and Cockburn, 1995). In contrast, intrasexual competition in superb starlings does not appear to be stronger in females than in males, perhaps resulting from equal sex ratios in social groups or other differences in group composition. Rather, our results support the idea that, despite higher variance in reproductive success among females in cooperative species, female-biased sexual dimorphism (i.e., role-reversal) is usually absent in cooperatively breeding species (Young and Bennett, 2013).

The high levels of within-bird similarity in songs and calls suggest that both types of vocalizations carry a strong signal of individual identity, and may therefore provide a basis for recognition. Although we have shown previously that calls appear to play a role in both individual and group recognition (Keen et al., 2013), the signal of identity in song may serve different purposes due to the specific behavioral contexts in which song is used. Flight calls are often made when entering and leaving a nest or entering a group territory, likely to identify the signaler as a specific individual or group member to prevent agonistic interactions or to encourage cooperation. In contrast, song is produced when perching in the group territory both while alone and in groups (Pilowsky and Rubenstein, 2013). In addition to helping to attract or compete for mates, song displays may be used in establishing social rank, as signaling identity may help to maintain within-sex dominance structures, since such hierarchies are only possible if identities are known (Barnard and Burk, 1979). Furthermore, signals of identity are expected to evolve in systems where individuals have repeated competitive interactions (reviewed in Tibbetts and Dale, 2007), as is the case in superb starling social groups. Because signaling identity while singing may aid in social competition, we hypothesize that this aspect of song is shaped by social selection and could help mediate the competition for social rank in both sexes.

Unlike the pattern observed in flight calls (Keen et al., 2013), song motifs from individuals in the same social group were not significantly more similar to one another than to song motifs from individuals in different groups. In other words, songs do not carry a detectable signal of group identity. This likely indicates that calls play a greater role in group recognition and maintaining boundaries between territories, and that calls may primarily serve to facilitate cooperation within social groups. Future studies should examine the social context in which these signals are produced, as this may offer further insight into the function of both signals.

Taken together, our results show that superb starling song is a complex signal that conveys information about identity and dominance rank. Although playback experiments are needed to demonstrate how this information is used, the present results support the idea that song is under both social and sexual selection to simultaneously express multiple traits (Gil and Gahr, 2002). Additionally, we show that the strength of selection on song diversity and the degree of individuality in songs is similar in males and females, suggesting similar strengths of selection. This may be due to mutual mate choice and high levels of intrasexual competition in both sexes, which is closely linked to the structure of superb starlings’ complex social system. Thus, song is likely to be used both in mate attraction and in dominance interactions that influence social rank; these two functions are linked, as dominant individuals are more likely to become breeders (Rubenstein, 2016). Our findings add to a growing body of work suggesting that song can function in both sexual and social contexts, and that the same selective forces can drive trait elaboration in both sexes in cooperatively breeding species.

## Supplementary Material

Supplementary Material 1

## Figures and Tables

**TABLE 1 |:** Estimate, standard error (SE), degrees of freedom (DF), *t*-values, and *p*-values for each term used in our selected GLMM (in bold print in Table S1).

Variable	Estimate	SE	DF	t	P

Status	0.18	0.087	22.56	2.01	0.056
Proportion	−0.31	0.13	21.96	−2.49	0.021
seasons breeding					
Sex	0.025	0.062	21.24	0.40	0.69
Age	0.028	0.012	19.82	2.37	0.028
