# Supplementary material for: Song in a Social and Sexual Context: Vocalizations Signal Identity and Rank in Both Sexes of a Cooperative Breeder
Source: Front Ecol Evol. Author manuscript; Available in PMC 2024 Feb 22. (PMC10883081; doi:10.3389/fevo.2016.00046)
Supplement: Supplementary Material 1 [file NIHMS1962403-supplement-Supplementary_Material_1.pdf]

Supplementary Material

Table S1. Alternate generalized linear mixed models (GLMMs) considered to measure the effect of age, sex, status (breeder or non-breeder) and proportion of adult seasons spent breeding (pbreed) on song diversity. Main effects in the model were chosen a priori based on the hypotheses of the study. The alternate models listed below were tested in order to determine whether to include interactions between these effects. More complex models that included interactions had larger values of Akaike's information criterion (AICc) and were rejected in favor of the more parsimonious model. ΔAICc values refer to the difference with the selected model (in boldface).

Model	AIC	DF	AICc	ΔAICc
**age+sex+status+pbreed**	**-17.34**	**7**	**-11.744**	**0**
age+sex*status+pbreed	-16.07	8	-8.49	3.25
age+sex+status*pbreed	-15.62	8	-8.04	3.70
age+sex*status*pbreed	-13.26	11	3.24	8.50

Table S2. Key to superb starling call and song motifs. All motifs which are found in calls are also found in songs. Spectrograms were created using Raven Pro 1.2 (Cornell Lab of Ornithology, Ithaca, NY, U.S.A.). Key was compiled by J.A.P.

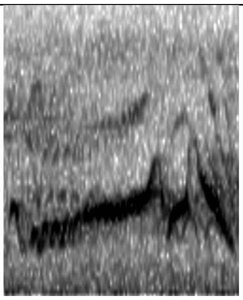 Flight Call 1: Up-trill N	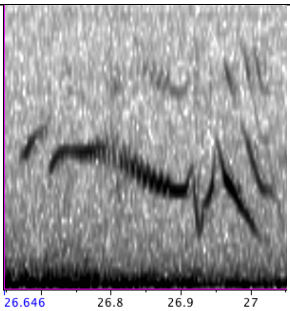 Flight Call 2: Down-trill N	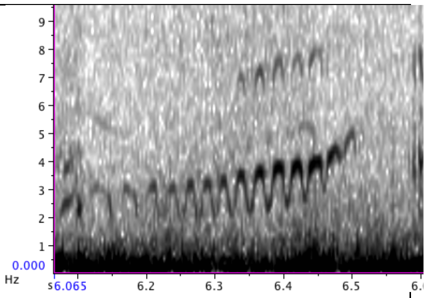 Flight Call 3: Long Trill
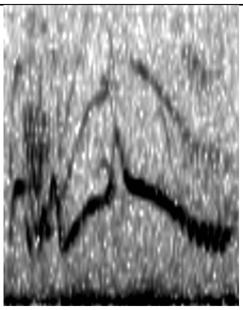 Flight Call 4: Squiggle N	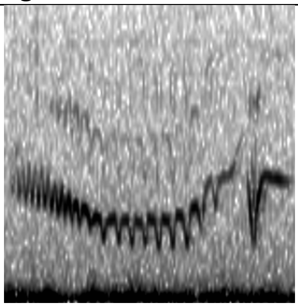 Flight Call 5: Adorned long trill	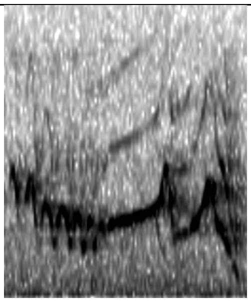 Flight call 6: Jagged long trill
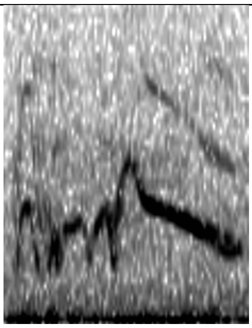 Flight Call 7: Squiggle arc	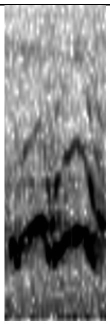 Flight call 8: Curly M	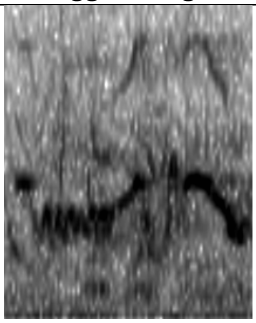 Flight call 9: Flat M
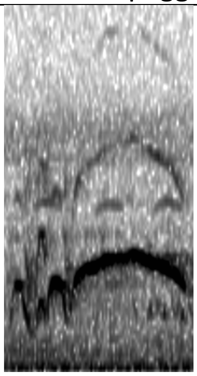 Flight call 10: Squiggle low curve	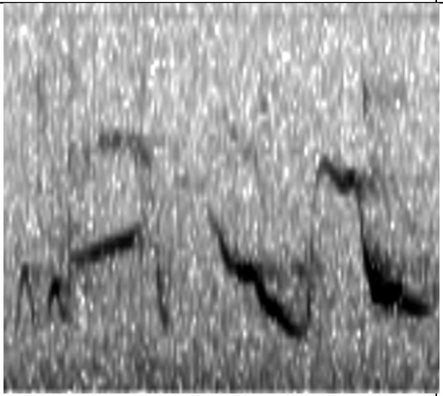 Flight call 11: M - N	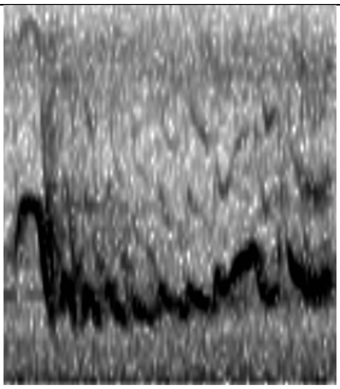 Flight Call 12: Hooked up-trill N

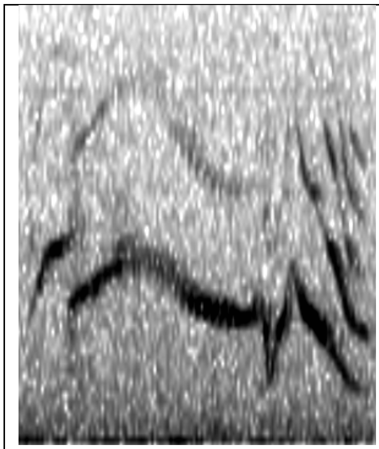 Flight call 13: Raised-trill N	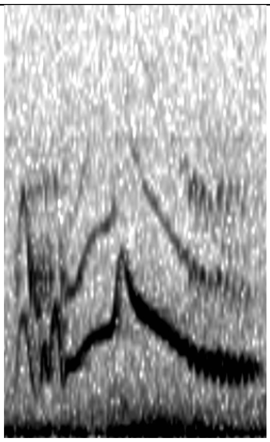 Flight call 14: Squiggle N trill	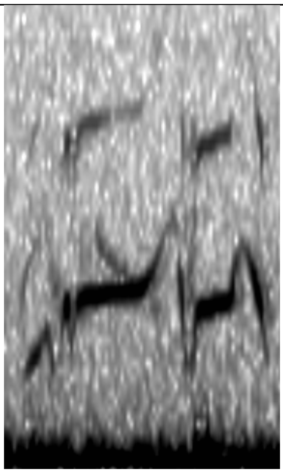 Flight Call 15: Squiggle M
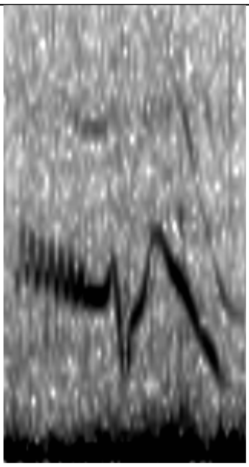 Flight call 16: Down-slope N	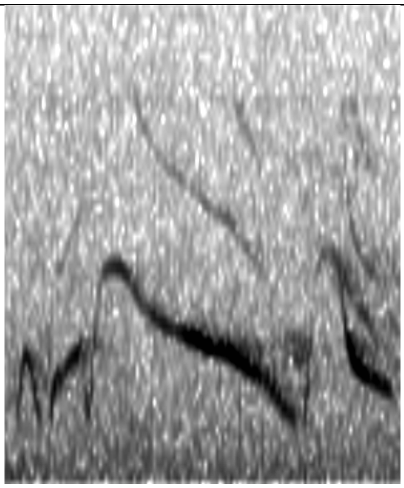 Flight call 17: N-N	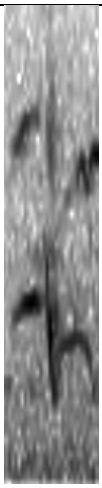 Flight call 18: Tilted M
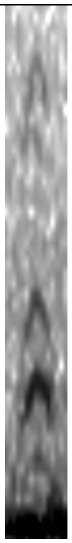 Syllable A	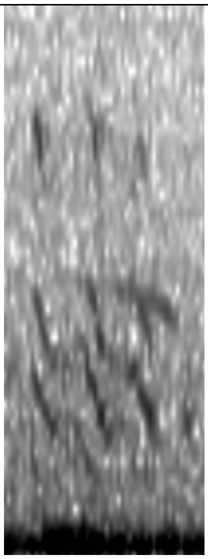 Syllable B, subtype 1	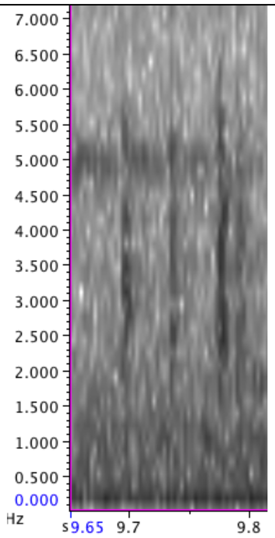 Syllable B, subtype 2

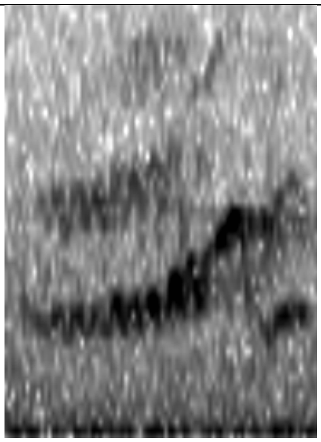

Syllable C

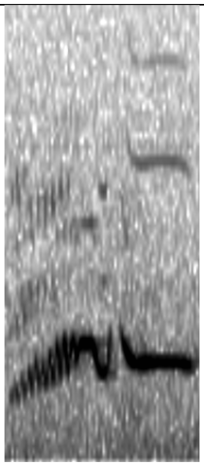

Syllable D

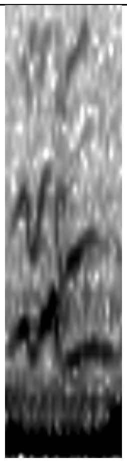

Syllable E

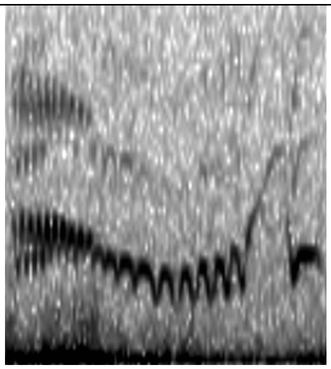

Syllable F

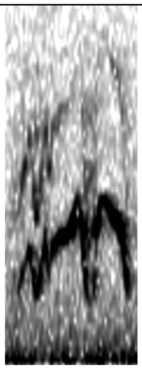

Syllable G, subtype 1

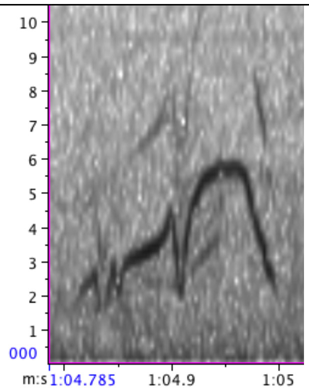

Syllable G, subtype 2

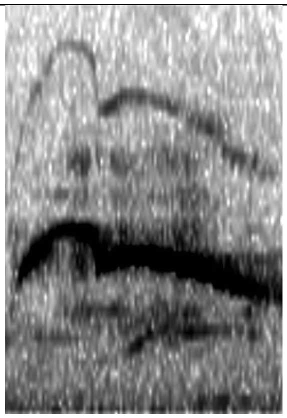

Syllable H

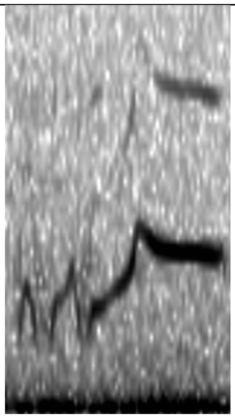

Syllable I, subtype 1

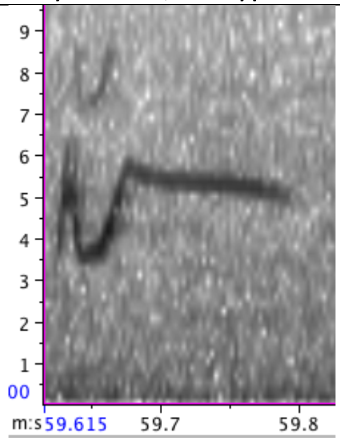

Syllable I, subtype 2

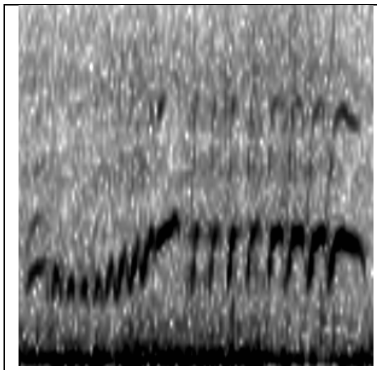 Syllable J	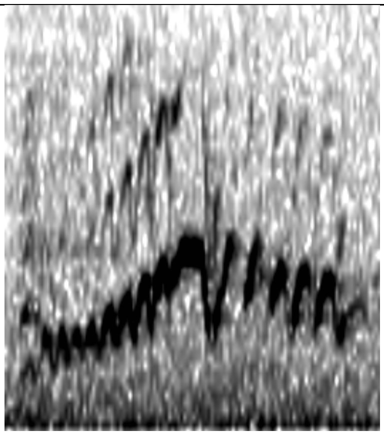 Syllable K	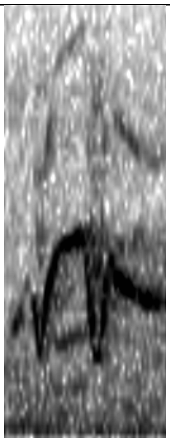 Syllable L
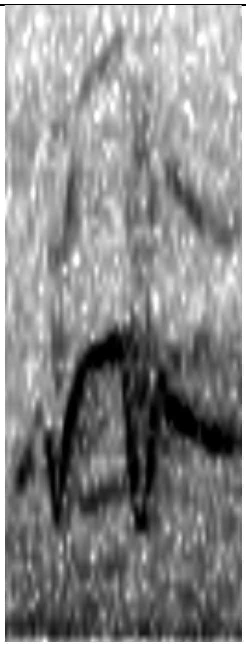 Syllable M	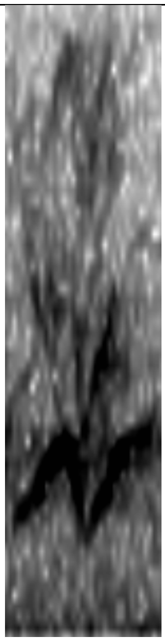 Syllable N	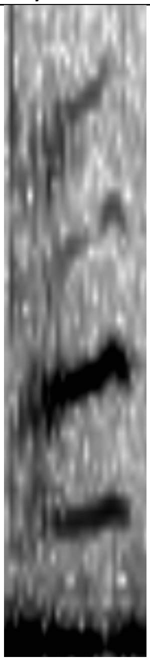 Syllable O
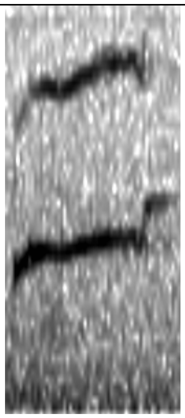 Syllable P, subtype 1	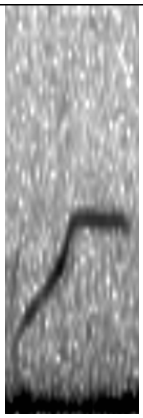 Syllable P, subtype 2	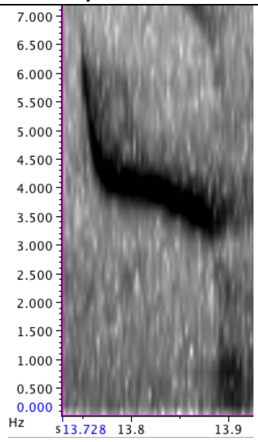 Syllable P, subtype 3

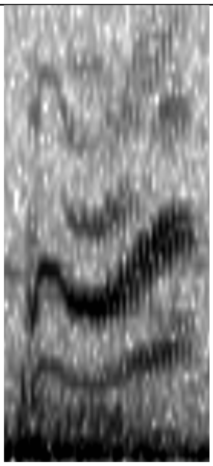<br>Syllable Q	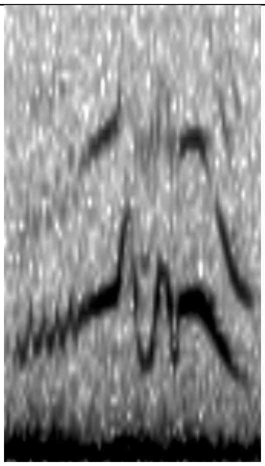<br>Syllable R	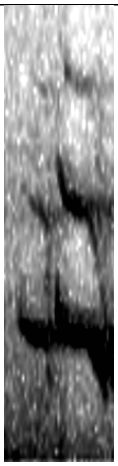<br>Syllable S
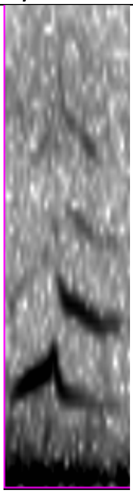<br>Syllable T	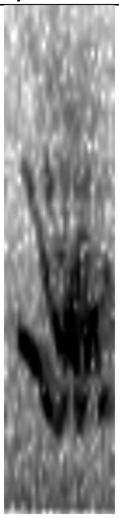<br>Syllable U	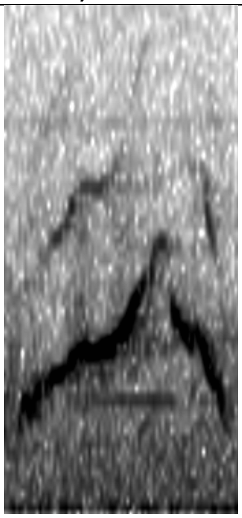<br>Syllable V
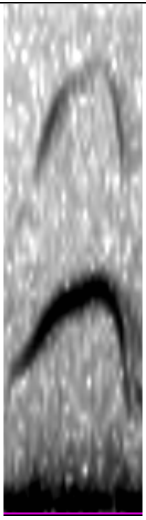<br>Syllable W	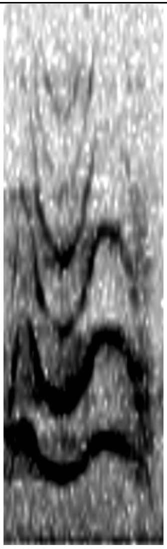<br>Syllable X	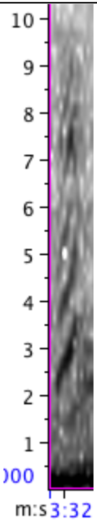<br>Syllable Y

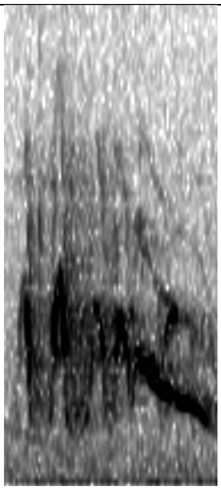

Syllable Z

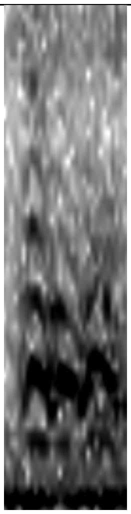

Syllable AA

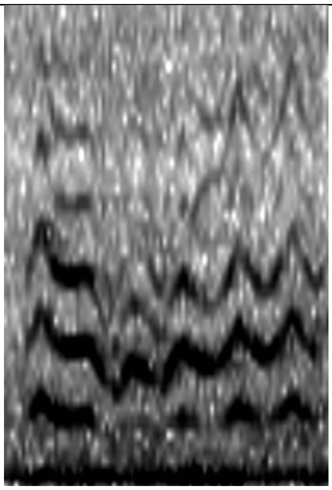

Syllable AB

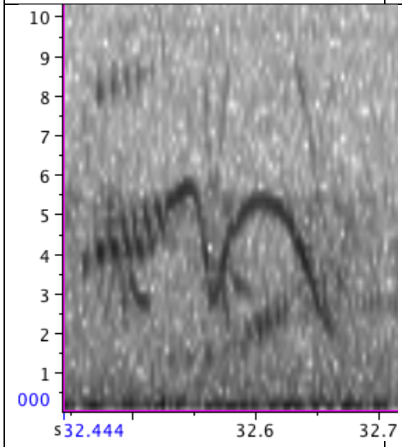

Syllable AC, subtype 1

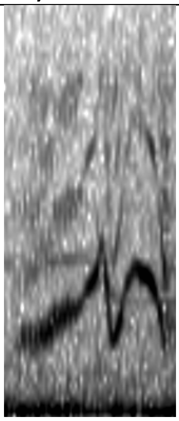

Syllable AC, subtype 2

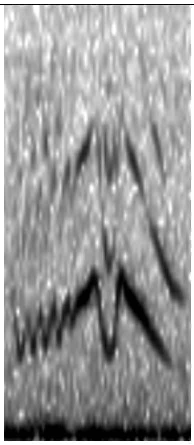

Syllable AC, subtype 3

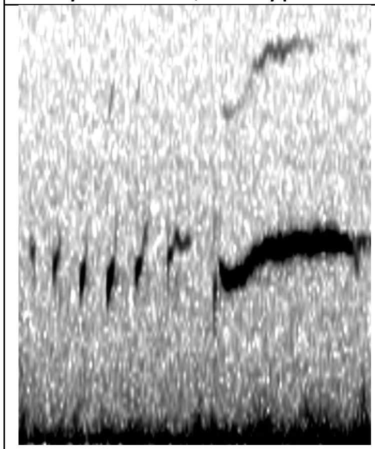

Syllable AD

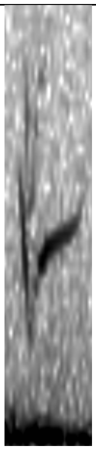

Syllable AE

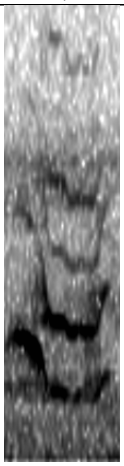

Syllable AF

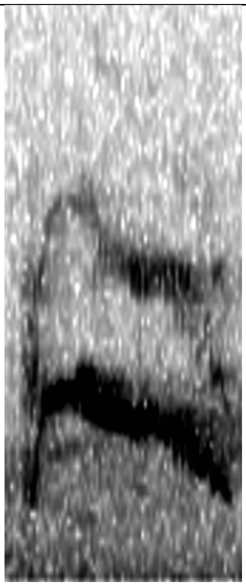 Syllable AG	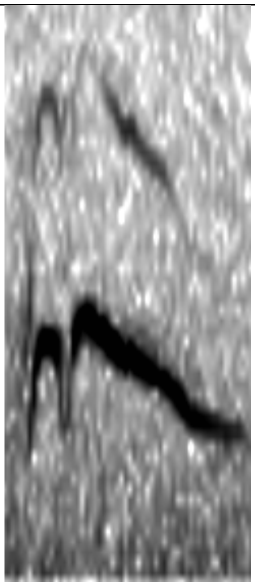 Syllable AH	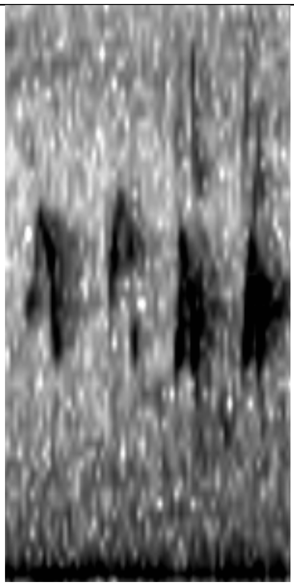 Syllable AI
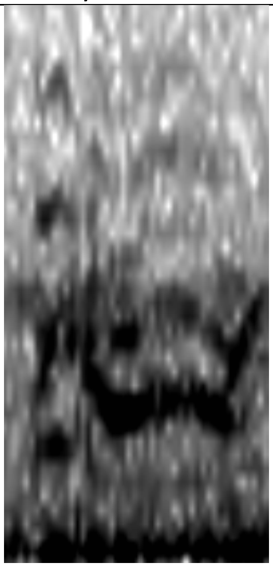 Syllable AJ	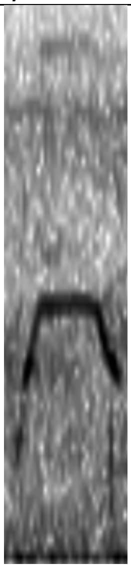 Syllable AK	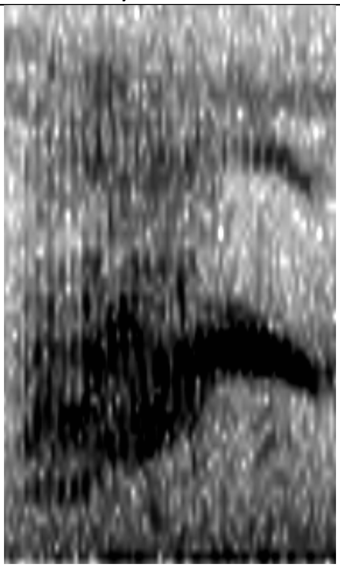 Syllable AL
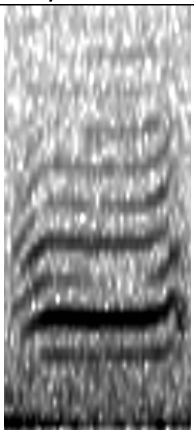 Syllable AM, subtype 1	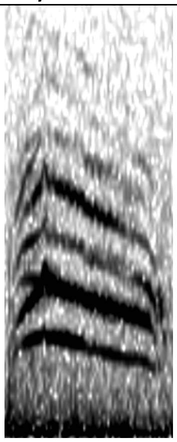 Syllable AM, subtype 2	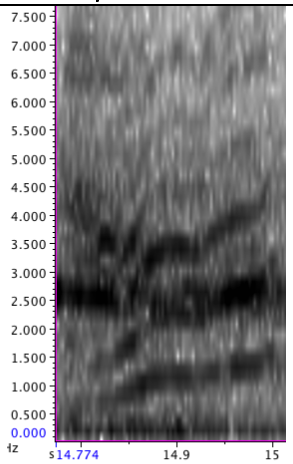 Syllable AM, subtype 3

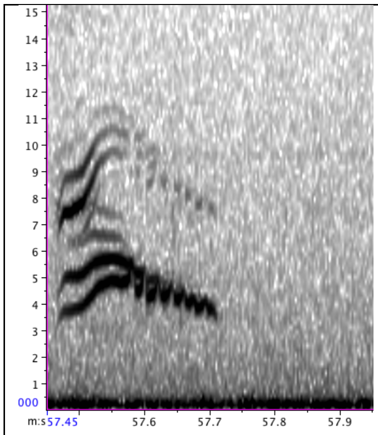

Syllable AN

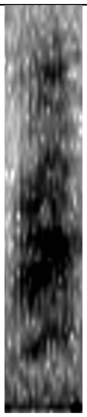
Syllable AO

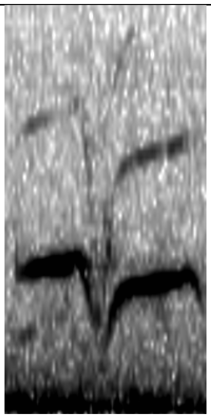
Syllable AP

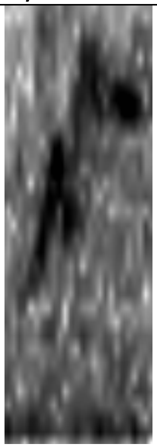
Syllable AQ

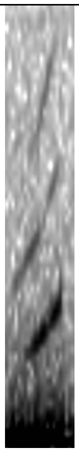
Syllable AR

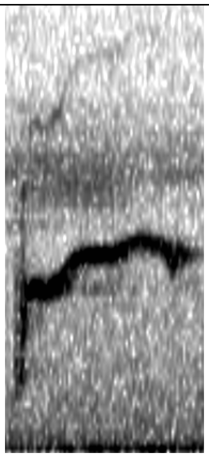
Syllable AS

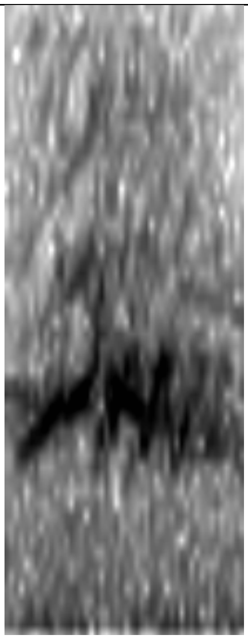
Syllable AT

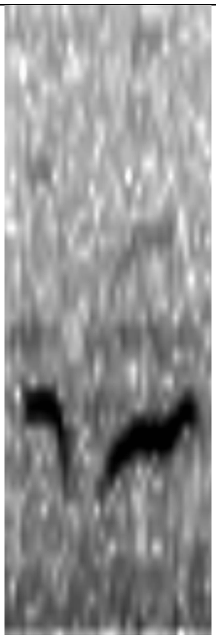
Syllable AU

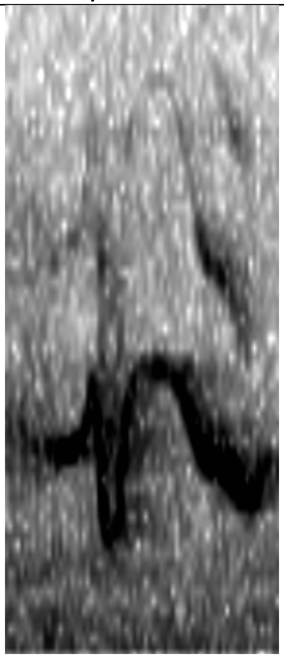
Syllable AV

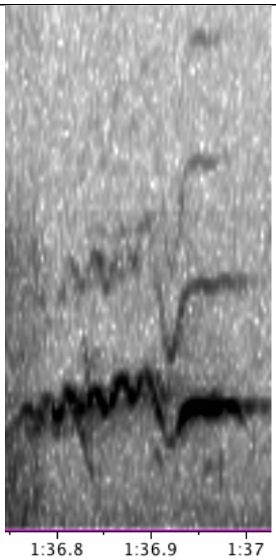

Syllable AW1

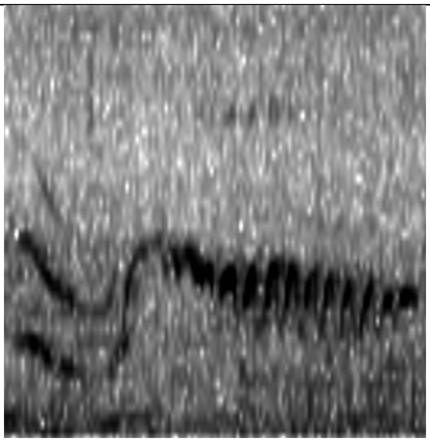

Syllable AW2

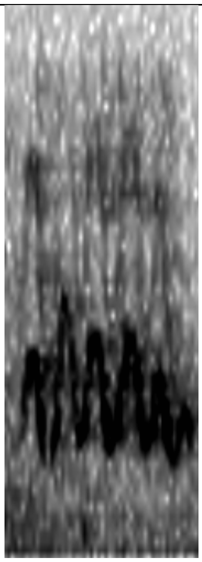

Syllable AX

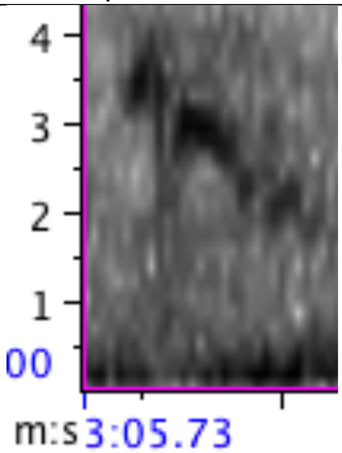

Syllable AY, subtype 1

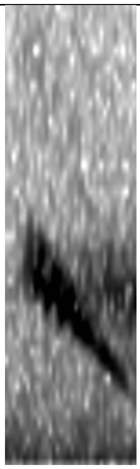

Syllable AY, subtype 2

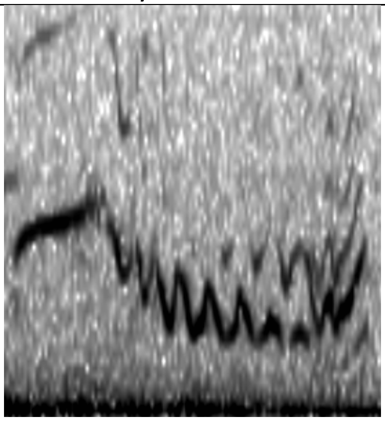

Syllable AZ

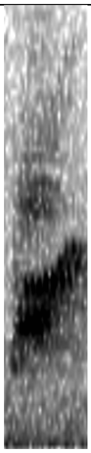

Syllable BA

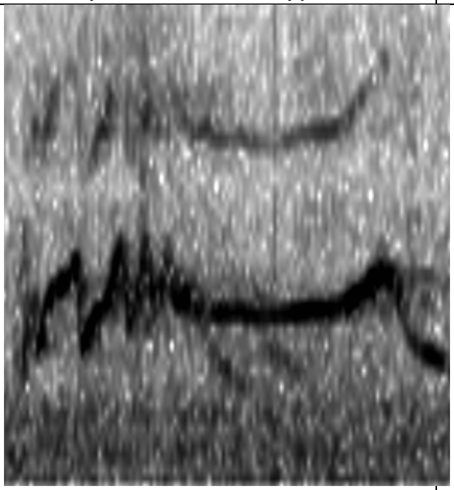

Syllable BB

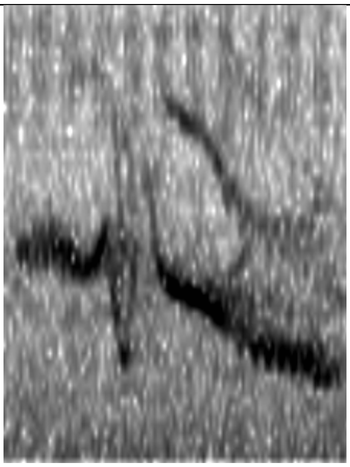

Syllable BC

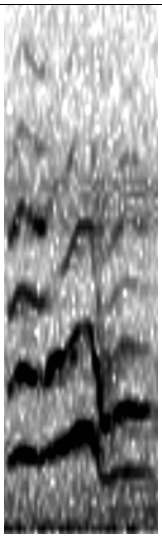<br>Syllable BD	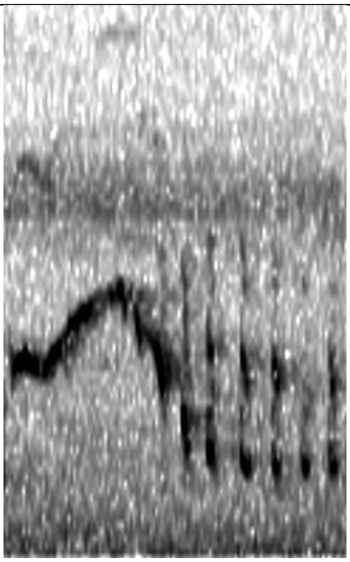<br>Syllable BE	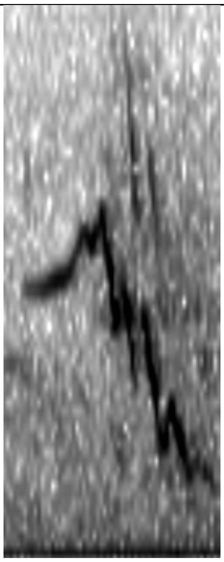<br>Syllable BF
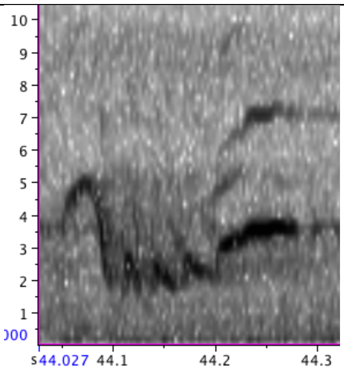<br>Syllable BG, subtype 1	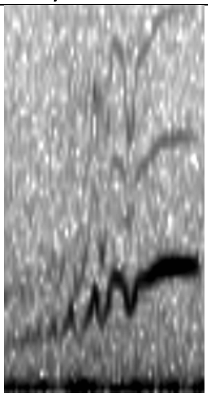<br>Syllable BG, subtype 2	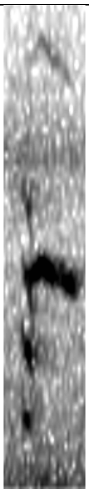<br>Syllable BH
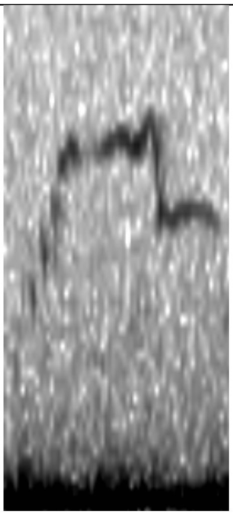<br>Syllable BI	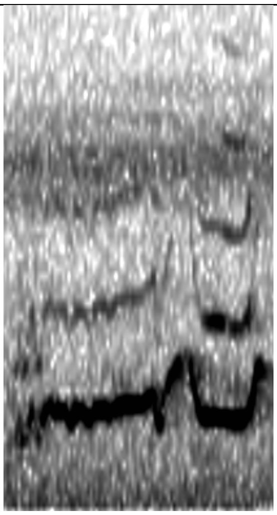<br>Syllable BJ	
